# Shining Light on Molecular Mechanism for Odor-selectivity of CNT-immobilized Olfactory Receptor

**DOI:** 10.1038/s41598-018-26105-0

**Published:** 2018-05-18

**Authors:** Liyun Zhang, Yuan Yuan, Tian Ren, Yanzhi Guo, Chuan Li, Xuemei Pu

**Affiliations:** 1 College of Chemistry, Sichuan University, Chengdu, 610064 P.R. China; 2 College of Management, Southwest University for Nationalities, Chengdu, 610041 P.R. China; 3 College of Computer Science, Sichuan University, Chengdu, 610064 P.R. China

## Abstract

Olfactory receptor (OR)-based bioelectronic nose is a new type of bio-affinity sensor applied for detecting numerous odorant molecules. In order to elucidate the effect of the adsorption of nanomaterial carriers on the receptor structure and its selectivity to odors, we used a systematic computation-scheme to study two OR models immobilized onto carbon nanotube. Our result indicates that there is a multistep OR-adsorption process driven by hydrophobic interaction. Many allosteric communication pathways exist between the absorbed residues and the pocket ones, leading to a significant shrinkage of the pocket. Consequently, the size-selectivity of the receptor to the odors is changed to some extent. But, the odor size and its hydrophobicity, rather than specific functional groups of the odor, still play a determinant role in binding OR, at least for the 132 odors under study. Regardless of the limitation for the odor size in initial recognition, the different-size odors could induce significant changes in the pocket conformation so that it could better match the pocket space, indicating the importance of the ligand-fit binding. Due to the CNT-induced shrinkage of the pocket, the CNT immobilization could increase the binding affinity through enhancing van der Waals interaction, in particular for the large odors.

## Introduction

The receptor biosensor is a new type of bio-affinity sensor, which is based on membrane receptor protein as a molecular recognition element. G protein-coupled receptors (GPCR)-based biosensors^1–3^ have attracted increasing research interests, which already can detect and discriminate a large number of chemical substances in complicated environments with high performances. As revealed, more than 350 human genes would encode functional olfactory receptors (ORs), which constitute the largest subgroup of GPCR family^4^. As the most fundamental elements in smell sensory neurons, ORs greatly contribute to high performance olfactory system and could recognize various odor molecules involved in different chemical classes, for example, alcohols, aldehydes, ketones, carboxylic acids, sulphur-containing compounds etc. Consequently, ORs are attractive and promising for the odorant detection in food security, environmental safety monitoring, drug screening and agricultural diagnosis5. Thus, many efforts have been devoted to developing OR-based biosensors^5–9^. Since nanomaterials possess unique electrical, mechanical and surface properties for the receptors loading^6–9^, various OR-based bioelectronic noses were constructed mainly through OR-conjugated to carbon nanotube transistors^6^, graphene micropatterns^7^, polypyrrole nanotubes^8^, plasma-treated bilayer graphene^9^, etc. These bioelectronic noses have been applied to detect numerous volatile odorant molecules, some of which exhibited high-performance. As known, the integrity and functionality of the receptors are most concerned in the fabrication of OR-based biosensors, which require to maintain natural conformations and native functions of the receptors in order to better recognize their natural ligands with low production costs and long using period. Despite many efforts devoted to the olfactory biosensors on experiments, it is difficult for the experimental techniques to detect subtle conformational variations in crucial regions of the receptors induced by the nanomaterial carriers, which play an important role in influencing their functions. Thus, it is highly desired to introduce some other techniques to detect the structural variations at microscopic level. In addition, it was found that one OR can recognize multiple odorants and one odorant is also recognized by multiple ORs^10^. Consequently, there have long been controversies in the selectivity of the ORs to the odors^11–13^, for example, the number of carbon atoms, the type of functional group, and other properties of the odors. The problems above significantly disfavor optimum design of the biosensor and limit its efficient-applications.

Molecular dynamics (MD) simulations have been widely applied to reveal the interaction between nanomaterials and proteins at the atomic level. Federica De Leo^14^ performed all-atom MD simulations to investigate structural and dynamic properties of monoclonal Cetuximab antibody (Ctx) adsorbed on a CNT surface and found that the immobilization would not damage its recognition ability. Binquan Luan^15^ used large scale all-atom molecular dynamics simulations to reveal that the graphene nanosheet could interrupt hydrophobic protein-protein interactions. Also, MD simulations were utilized to investigate the interactions of single-walled CNTs with enzymes^16^, amino acids^17^, kinds of peptides^18^, and polysaccharides^19^. However, these MD works were almost performed within several hundred nanoseconds and did not consider further adsorption possibly occurring at longer time.

Since X-ray crystal structures of the olfactory receptors have been absent and the sequences of the ligand binding site are low conservation, theoretical researches on the receptors have been very limited. Furthermore, these works mainly focused on interactions between some odors and the free receptors based on several homology models^20–22^. To our best knowledge, there has been a lack of computational works on interactions between the carbon nanomaterial and the olfactory receptors. Recently, Park *et al*. crystallized and characterized one active conformation of the retinal-free rhodopsin apoprotein (opsin), which presents similar properties to the olfactory receptor like the binding capability to hydrophobic ligands and the H-bond patterns between the ligands and the receptor. Thus, it was considered to be a promising structural model for the olfactory receptor^23^. Ilia A. Solov’yov^24^ already took it as the model of the olfactory receptor to study its dynamical behavior and electron-phonon coupling with some odorants using MD method.

Based on all the consideration above, we, in the work, used microsecond timescale MD simulation and virtual screening methods to explore the effect of the CNT adsorption on the structure of the olfactory receptor and its selectivity to the odors, in order to address on the problems above. Herein, we selected two olfactory receptor models in order to obtain more systematic information. One is a specific homology model of human olfactory receptor hOR2AG1, which have been widely used in experimental studies for the olfactory biosensors^7–9^. The other is the recently crystallized opsin. Our results reveal the CNT-adsorption induced changes in the structure of the OR receptor (in particular for the ligand-binding pocket) and the selectivity to the odorants. In addition, we also identify some important structural pathways, residues and interaction forces contributed to the change, finally elucidating their molecular mechanisms. These observations from the work could provide helpful information at the molecular level for structural modification and application of the olfactory biosensors.

## Results and Discussions

We selected two models to construct the structure of the olfactory receptor in the work. One is rhodopsin apoprotein (abbreviated as opsin) and the other is the homology model of hOR2AG1 (abbreviated as hOR2AG1). The opsin and the hOR2AG1 were individually adsorbed onto one single-walled CNT, leading to the two receptor-CNT complex systems, which are labelled as CNT-opsin and CNT-hOR2AG1, respectively. As a reference, the two free olfactory receptor systems were also studied, which are named as free-opsin and free-hOR2AG1. An ensemble strategy was proposed in the work. First, MD simulations with one microsecond scale time were performed for the four different systems mentioned above, in order to observe the adsorption process and the structure change of the receptor. Then, the lowest energy frame from the last nanosecond of the 1 μs trajectories for the opsin models in immobilized state and in free one were selected as representatives to conduct virtual screening to one ligand set consisted of 132 representative odors, in order to probe the effect of the CNT adsorption on the selectivity of OR to the odors. To elucidate the molecular mechanism of the selectivity, further 100-ns MD simulations were performed for three representative complexes, which involve in three odors with different molecular sizes.

### Adsorption process and its effect on the structure of the olfactory receptor

**Figure 1:**
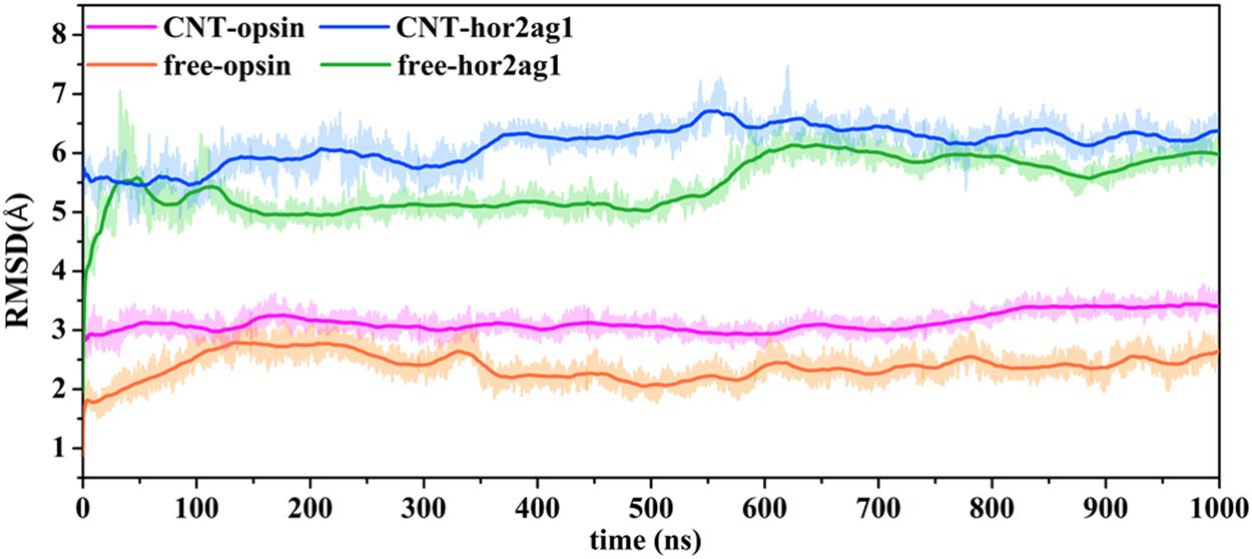
RMSD values of backbone atoms for the two OR models during the 1000 ns simulation time. The RMSD values are deviations from the crystal structure for the opsin model and from the initial homology structure for the hOR2AG1 model.

In order to observe the role of the CNT-immobilization in influencing the entire structure of the receptor, root-mean-square deviations (RMSDs) of backbone atoms for all the OR systems under study were calculated and the result is shown in Fig. 1. The results show that the RMSD values of the four systems approach to convergence during the last several hundred nanoseconds. The adsorption induces significant variations on the structure of the receptor, as evidenced by larger RMSD values of the immobilized receptor than that of the free one. Compared to the free receptors, the immobilized ones present smaller RMSD fluctuations, implying enhanced rigidity upon CNT-immobilization and supporting the experimental observations that the immobilization could improve the stability of proteins to some extent^25,26^. In addition, the RMSD values of the hOR2AG1 homology model are significantly larger than those of the opsins both for the CNT-immobilized type and the free one, which should be attributed to instability of the homology model.

**Figure 2:**
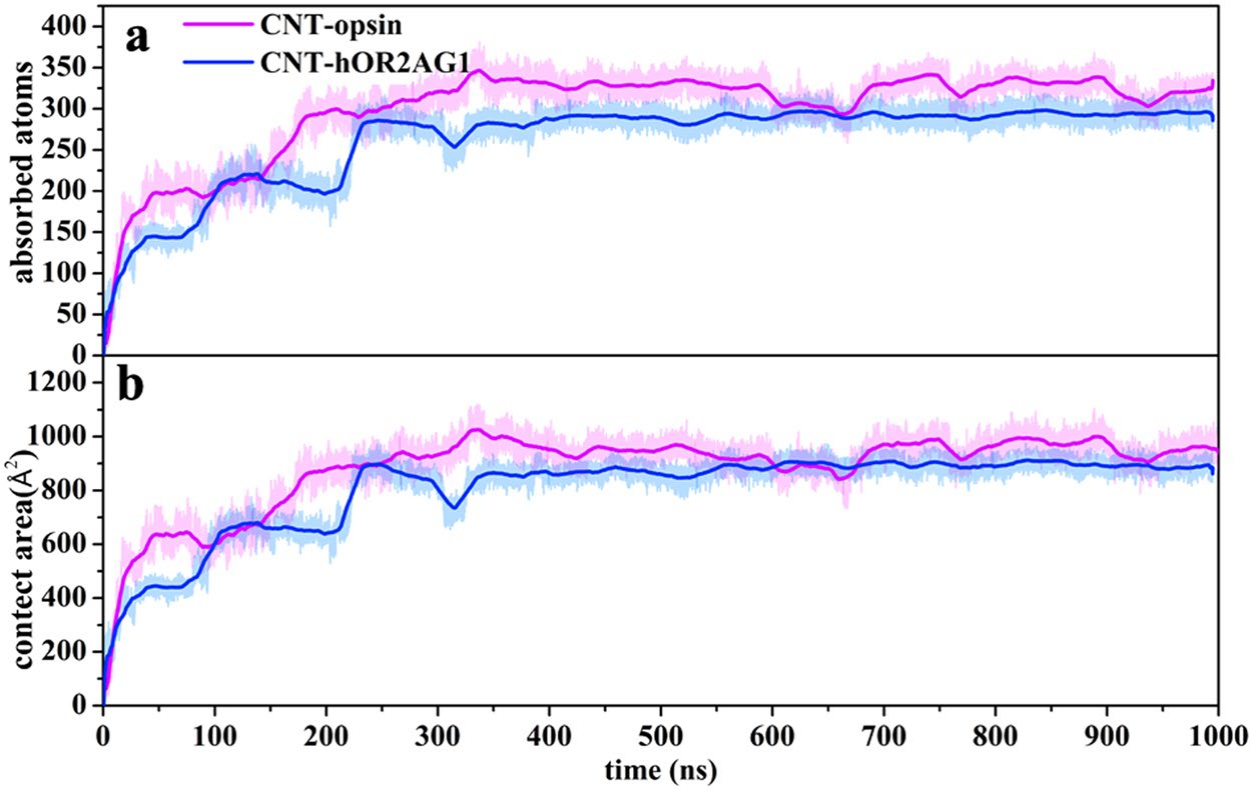
The adsorption process of the two OR models. (**a**) The number of atoms adsorbed by CNT and (**b**) the contact area between CNT and OR during the 1000 ns simulation time.

To gain insight into non-covalent adsorption behaviors for the two receptor models, we monitored changes in the number of non-hydrogen atoms adsorbed onto the CNT surface over the whole simulation time in terms of a criterion of 6 Å, which is generally considered as the distance range of hydrophobic interaction^27^. In terms of equation (1), we also calculated the contact area between the CNT surface and the receptor to further evaluate the adsorbed extent. The two results are depicted in Fig. 2.

1 $${\rm{contact}}\,{\rm{area}}=\frac{1}{2}[(SA{S}_{{\rm{rec}}}+SA{S}_{{\rm{cnt}}})-SA{S}_{{\rm{complex}}}]$$

where *SAS*_rec_, *SAS*_cnt_, and *SAS*_complex_ denote the solvent accessible surface areas (SASA) of free receptor, CNT, and receptor-CNT complex, respectively.

**Figure 3:**
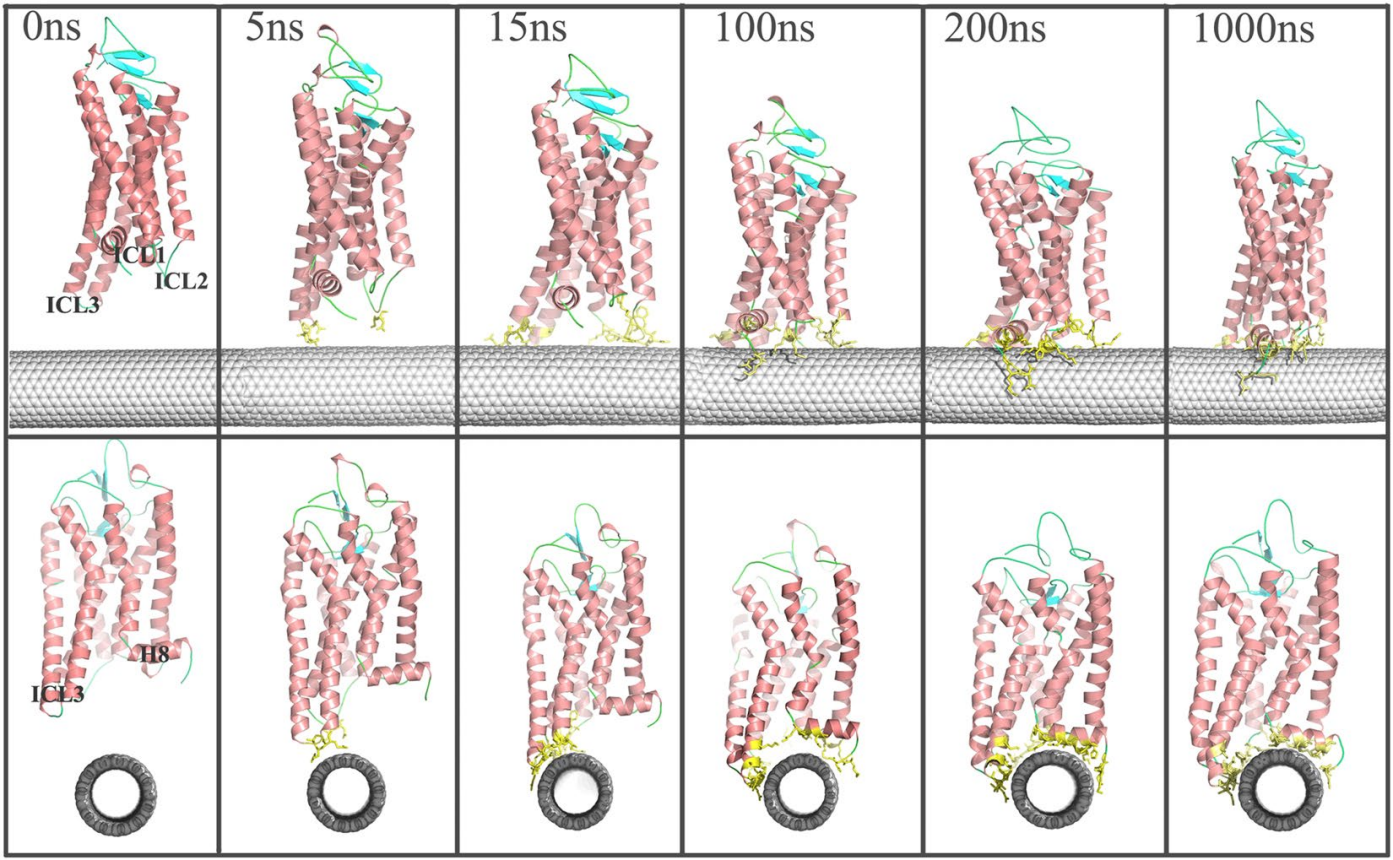
The front view (top) and side view (bottom) of the representative snapshots in the adsorption process for the opsin system. The snapshots are selected from the 1 μs simulation trajectory in terms of changes in the RMSD values and the adsorbed atoms. Opsin is displayed in cartoon style and colored by secondary structure type. CNT is displayed in CPK style. The residues within 6 Å of the CNT surface are displayed in yellow line style.

By comparing Fig. 2a and b, it is found that the change trend of the contact area is consistent with the number of adsorbed atoms, indicating that the main driving force to the adsorption is the hydrophobic interaction. In addition, in terms of the two calculated parameters, some representative snapshots were selected for the CNT-opsin system to visually view the variation of the receptor conformation upon adsorption (vide Fig. 3). It can be seen from Fig. 2 that there is a rapid and strong adsorption within first 15 nanoseconds for the two models. Figure 3 further reveals that the ICL3 region of the opsin first attaches to the CNT surface, and then other intracellular loops including helix 8 and carboxy-terminal tail gradually approach to CNT. After that, the adsorption process reaches a temporary plateau with about dozens of nanoseconds, in which no significant changes occur in the distance between the receptor and CNT. Then, the receptor continually adjusts its orientation to some extent and spread itself along the surface to further sufficiently interact with CNT during the following several hundred nanoseconds, as judged from Figs 2 and 3. After about 350 ns, the adsorption seems to reach relatively stable stage with ~320/280 adsorbed atoms and ~910/880Å^2^ contact area for the CNT-opsin and CNT-hOR2AG1 systems, respectively. In the stage, the receptor maximizes its interaction with the tube surface until 1000 ns. As shown in Fig. 2, the adsorption processes of the two models both present several jumps for the number of absorbed atoms and the contact areas. Previous theoretical studies on some typical proteins immobilized to graphene^28^ and CNT^27,29^ also showed the multistep adsorption. Different from short jump times revealed by the previous works, the multistep adsorption in the work covers a relatively long timescale, reaching several hundred nanoseconds.

### Key residues contributed to the affinity between the olfactory receptor and CNT

**Figure 4:**
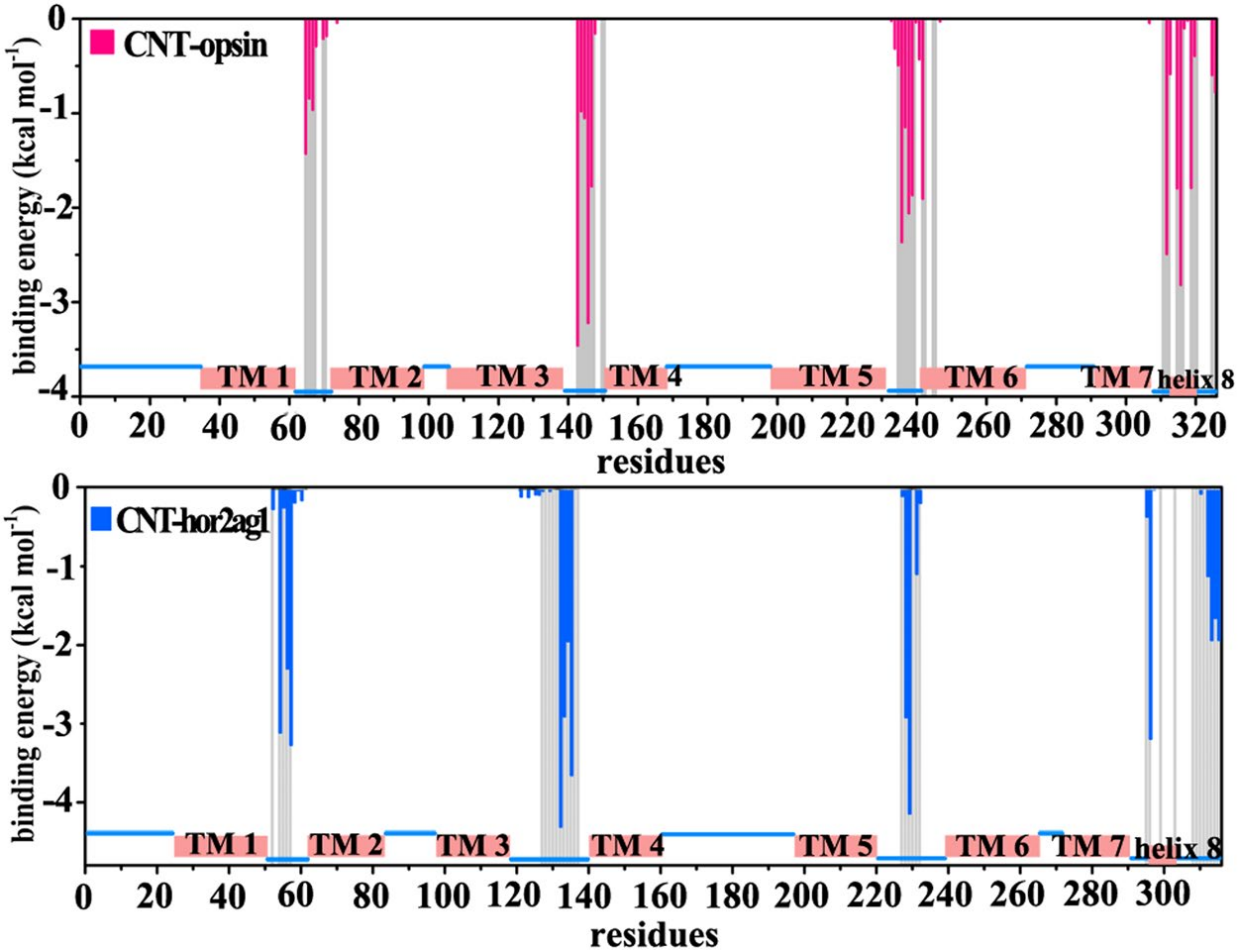
Per-residue contribution to the binding energy between CNT and OR. The residues located within 6 Å distance around CNT are also highlighted in gray bars.

To further identify crucial residues contributed to the affinity between the receptor and CNT, we used Molecular mechanics/Poisson–Boltzmann Surface Area (MM-PBSA) to calculate their binding energies based on the last 100 ns MD trajectories. As shown in Table 1, the binding energy of the CNT-opsin system (−121.8 ± 3.6 kcal mol^−1^) is slightly greater than that of CNT-hOR2AG1 (−115.8 ± 5.3 kcal mol^−1^), which is consistent with the contact area and the absorbed atoms above. The decomposition of the binding energy further confirms that the hydrophobic van der Waals interaction plays a main role in driving the adsorption. Figure 4 shows Per-residue contribution to the binding energy. It can be seen that the residues within a distance of 6 Å from the CNT surface (highlighted in gray bars in Fig. 4) present main contributions to the binding energy, implying the importance of the initially placed orientation between OR and CNT. As shown in Fig. 4, the adsorbed residues of the opsin distribute in intracellular ICL2 (Met143-Arg147), ICL3 (Gln236-Glu239, Thr342) and helix 8 (Gln312, Asn315-Cys316, Thr319). Nevertheless, the residues of the hOR2AG1 system with great contribution to the adsorption are mainly located at ICL1 (Arg54, His56-Met57), ICL2 (Tyr132-Leu135), ICL3 (Met228-Pro229, Asn231), helix 8 (Lys295-Val297) and C-terminal (Ala312-Leu316).The two receptor models present to some extent differences for the adsorption region. But, the important residues contributed to the adsorption all involve in ICL2, ICL3 and the helix 8 for the two models. Recently, it was reported that some residues of the olfactory receptor located at the helix 8 play a functional role in response to the ligand binding^30^. In addition, it was also indicated that the intracellular regions of some A class GPCRs involve in an allosteric binding site, which could influence the recognition of orthosteric ligands^31,32^. These observations imply that the adsorption of some residues in the helix 8 and the intracellular loops should induce to some extent functional variations in the ligand binding. Thereby, we further analyze the structure change in the ligand binding pocket and the allosteric pathway from the adsorbed residues to the pocket residues in the following.

**Table 1:** Components of the binding energy between the carbon nanotube and OR and their standard errors (in kcal mol^−1^).

Contribution	CNT-opsin	CNT-hOR2AG1
*ΔE*_ele_^a^	−0.0 ± 0.0	−0.0 ± 0.0
*ΔE*_vdw_^b^	−176.8 ± 4.5	−182.0 ± 6.9
*ΔE*_gas_^c^	−176.8 ± 4.5	−182.0 ± 6.9
*ΔG*_npsolv_^d^	−6.5 ± 0.2	−7.9 ± 0.3
*ΔG*_psolv_^e^	61.6 ± 2.8	74.1 ± 4.2
*ΔG*_solv_^f^	55.1 ± 2.9	66.2 ± 4.1
*ΔG*_binding_^g^	−121.7 ± 3.6	−115.8 ± 5.3

^a^Electrostatic energy.

^b^Van der Waals interaction energy.

^c^Total gas phase energy.

^d^Nonpolar solvation energy.

^e^Polar solvation energy calculated by solving Poisson–Boltzmann equation.

^f^Solvation energy.

^g^Binding energy.

Δ*E*_gas_ = Δ*E*_ele_ + Δ*E*_vdw_ + Δ*E*_int_, Δ*G*_solv_ = Δ*G*_npsolv_ + Δ*G*_psolv_, Δ*G*_binding_ = Δ*E*_gas_ + Δ*G*_solv_.

### Mechanism regarding conformational changes in the odor-binding pocket induced by the CNT adsorption

**Figure 5:**
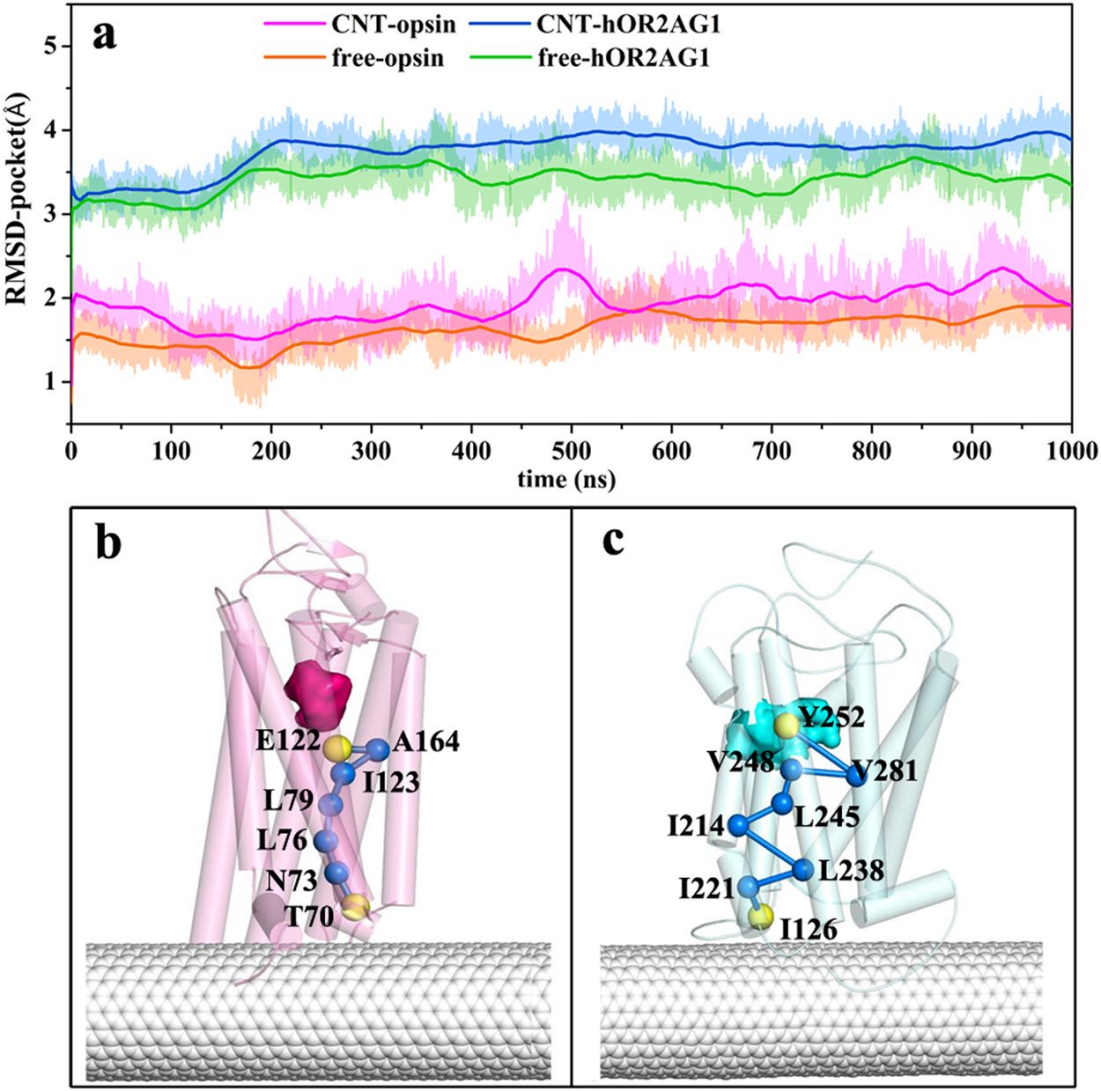
Structural changes in the odor-binding pocket induced by the CNT adsorption and allosteric communication pathways with the highest frequency. (**a**) RMSD values of the backbone atoms for the binding pockets of the two OR models (opsin and hOR2AG1) during the 1000 ns simulation times. The RMSD values are deviations from the crystal structure for the opsin model and from the initial homology structure for the hOR2AG1 model. The allosteric communication pathways with the highest frequency from the CNT absorbed residues to the pocket residues for the opsin system (**b**) and the hOR2AG1 system (**c**). The pathways were calculated using the last 100 ns trajectories of the 1 μs simulation time.

To gain insight into the effect of CNT-immobilization on the conformation of the ligand-binding pocket, we calculated RMSD values of the pocket residues, as shown in Fig. 5a. Similar to the total RMSD variation of the receptor, the RMSD values of the hOR2AG1 pocket are higher than those of the opsin model. In addition, the CNT-immobilized receptors for the two models show larger RMSD values compared to the free receptors. The average RMSD values of the binding pocket over the last 100 ns are 2.1 Å for the immobilized opsin and 3.8 Å for the immobilized hOR2AG1, while the values are 1.6 Å for the free opsin and 3.2 Å for the free hOR2AG1.

In order to elucidate the change mechanism, we utilized protein structure network (PSN) method to analyze the allosteric communications from the residues absorbed onto CNT (within a distance of 6 Å around CNT) to the pocket residues (vide Fig. 5b,c). Based on the inter-connectivity of the PSN nodes and the residue correlated motions, the shortest path was identified for the last 100 ns trajectories of the 1 μs MD simulations.

For the CNT-opsin system, the number of communication pathways with frequencies higher than 50% is 42, in which the absorbed residues T70, C316 and T320 are the top three most frequently starting nodes and the pocket residues E122, T118 and Y268 are the top three most frequently visited ending nodes. For the CNT-opsin system, the shortest path with the highest frequency (81%) between the two regions is shown in Fig. 5b, which starts from T70 of ECL1 and ends at the E122 residue located at TM3. The pathway is consisted of N73, L76, L79, I123, and A164, which involve in TM2, TM3, and TM4. For the CNT-hOR2AG1 system, it has much more communication pathways (98) with high frequencies (>50%), which may be attributed to more residues consisted of the ligand binding pocket than the CNT-opsin system. The pathway with the highest frequency starts from I126 and ends at Y252, which passes through the six residues I221-L238-I214-L245-V248-V281. The pathway involves in TM5, TM6, and TM7, different from the system of the CNT-opsin. The three absorbed residues (I126, R122, and A125) significantly contributed to the binding are frequently starting nodes and the frequently visited residues in the ligand-binding pocket are Y252, D112, and V199.

The observation from the PSN analysis indicates that many allosteric communications exist from the absorbed residues in the intracellular region to the ligand-binding one, which should contribute to the immobilization-induced change in the conformation of the ligand-binding pocket. In addition, some crucial residues in these allosteric pathways were identified. It can be assumed that the modification on these crucial residues like mutations or chemical modifications should significantly influence the selectivity or recognition of the receptors to odorant molecules.

### Effects of the CNT adsorption on the selectivity of the receptor to the odorants revealed by virtual screening

**Figure 6:**
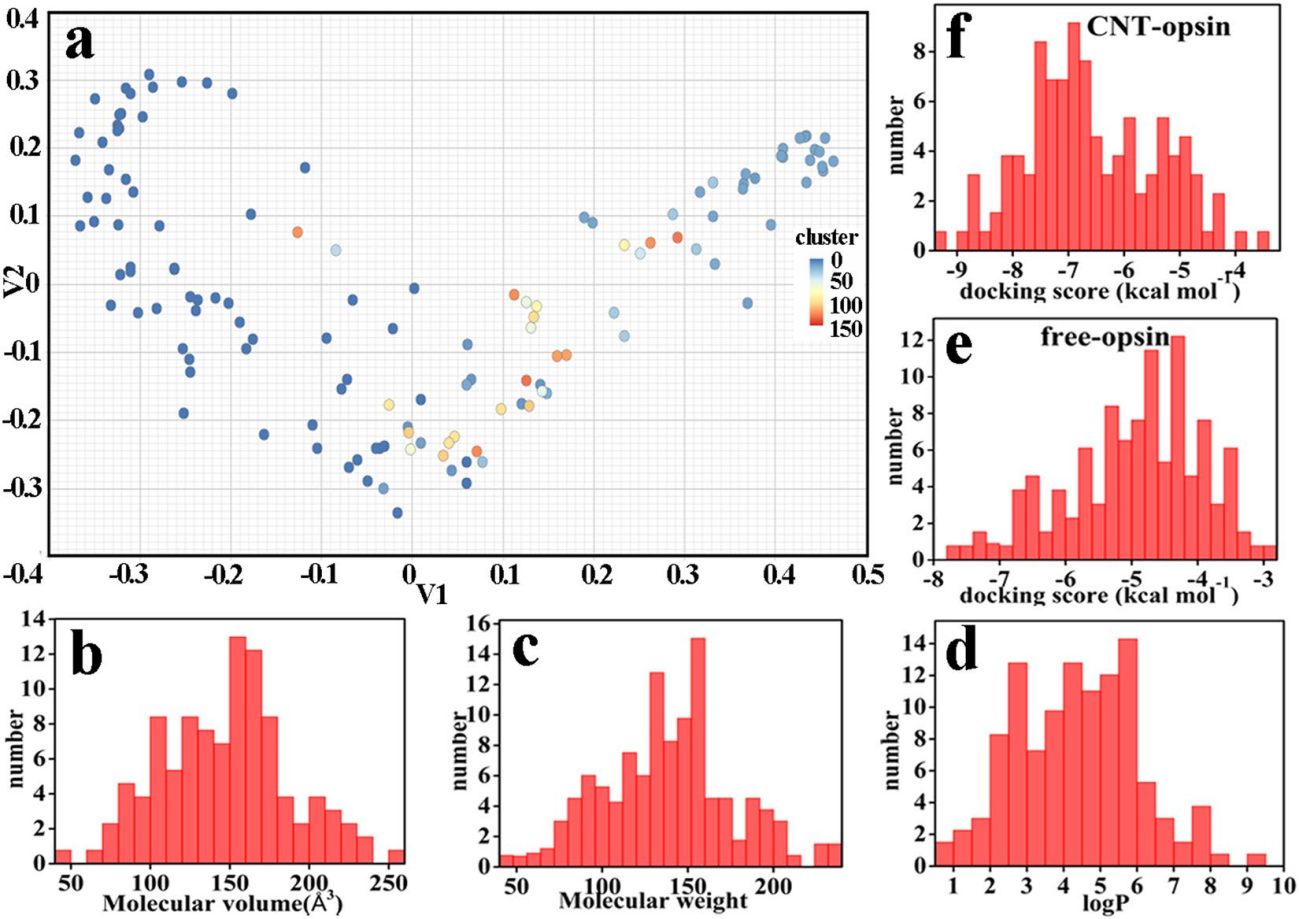
The distribution for molecular properties of the ligand set. (**a**) The projection the 132 odors into a 2-Dimensional plane in terms of their structural similarities. The populations of the odors with respect to (**b**) the molecular volume, (**c**) the molecular weight and (**d**) logP. The distributions of scores for (**e**) the free opsin and (**f**) the CNT-immobilized opsin.

In the part, we selected the opsin model as a representative to probe the effects of the CNT-immobilization on the selectivity of the olfactory receptor to the odors using virtual screening method, which has been widely applied for drug design^33,34^. We constructed a ligand set consisted of 132 small molecules. The 132 molecules were already proven to be mammalian and insect-specific odorants by some theoretical or experimental works^35–38^ and involve in different chemical classes like alcohols, ketones, acids, and aldehydes compounds. Figure 6a shows a projection of the 132 odorant molecules into a 2-Dimensional plane in terms of their structural similarities^39^. It can be seen that the molecular properties of the 132 odors are distinctly different. In addition, Fig. 6b–d also show the populations of the odors with differently physicochemical properties like the molecular weight, the molecular volume and logP. The distributions of binding energy scores for the free and CNT-immobilized opsins are also displayed in Fig. 6e,f. The results all show that the ligand set constructed is a non-biased sampling of the odorants involved in a wide range of molecular weight (42–236), volume (45–267 Å^3^), and logP (0.7–9.6) as well as the binding scores. Consequently, the ligand set is representative for the odorant molecules.

**Figure 7:**
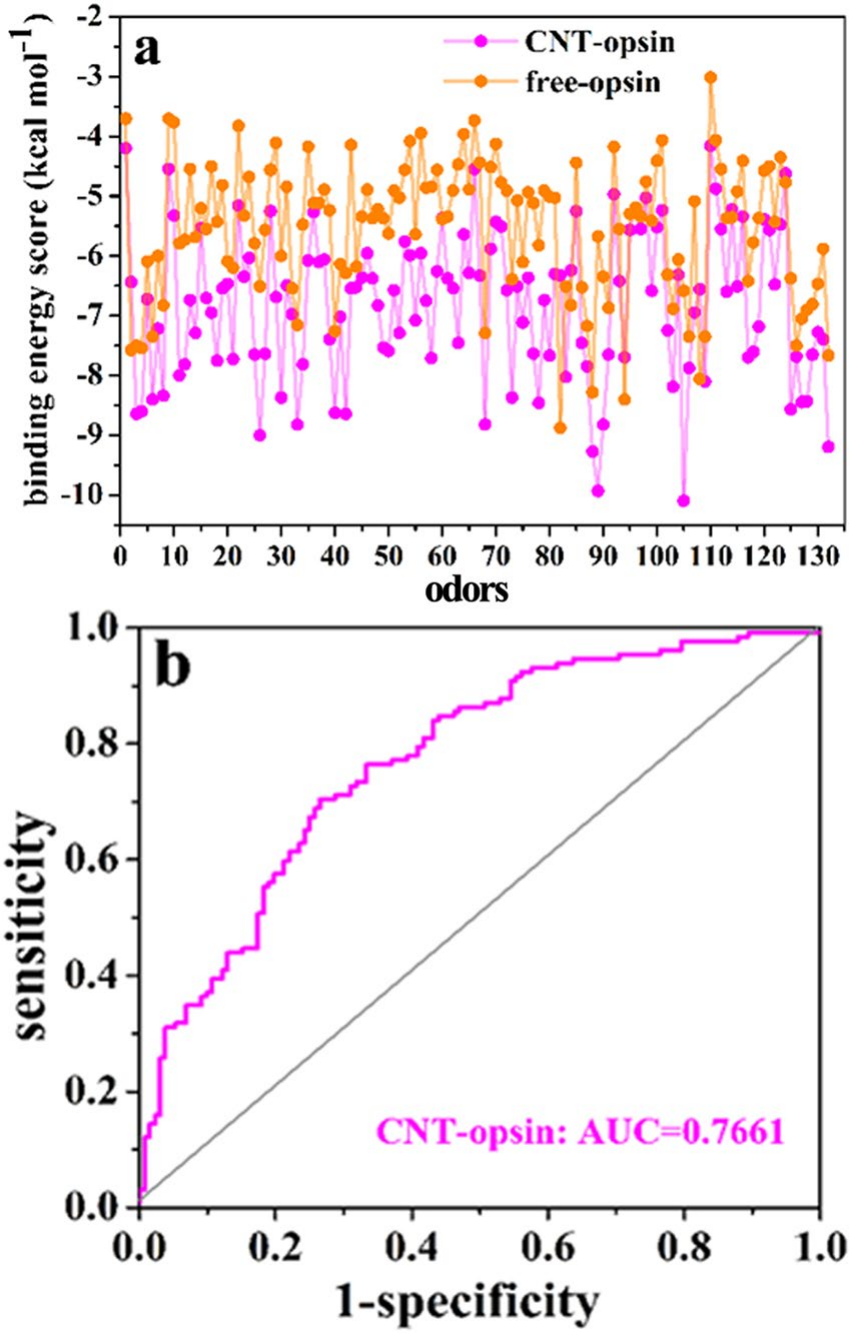
The screening results for the opsin-CNT and opsin-free systems. (**a**) The docking scores between the 132 odors and the opsin in the free type and the immobilized one. (**b**) The receiver operating characteristic (ROC) curve of the 132 odors to the immobilized opsin.

The screening results for the opsin-CNT and opsin-free systems are shown in Fig. 7. For the majority of the 132 odors, the binding energy scores of the CNT-immobilized opsin are significantly higher than those of the free one. Although there are no direct experimental evidences from the same system to validate our binding result, it was reported that zinc metal nanoparticles strongly enhance olfactory responses quantified by electroolfactogram (EOG) to some odorants like cyclohexanone, methyl benzoate, and acetophenone^40^, in line with our observation. In addition, the odors with relatively low binding energies are small molecules like acetaldehyde, ethanol, etc., as reflected by Fig. 7a. We plot the receiver operating characteristic (ROC) curve and calculate the area under ROC curve (AUC value) to characterize the screening performance of the 132 odors to the immobilized and the free opsins (vide Fig. 7b). It is clear that the high screening performance of the odors is presented for the CNT-immobilized opsin, as evidenced by 0.7661 of AUC.

**Figure 8:**
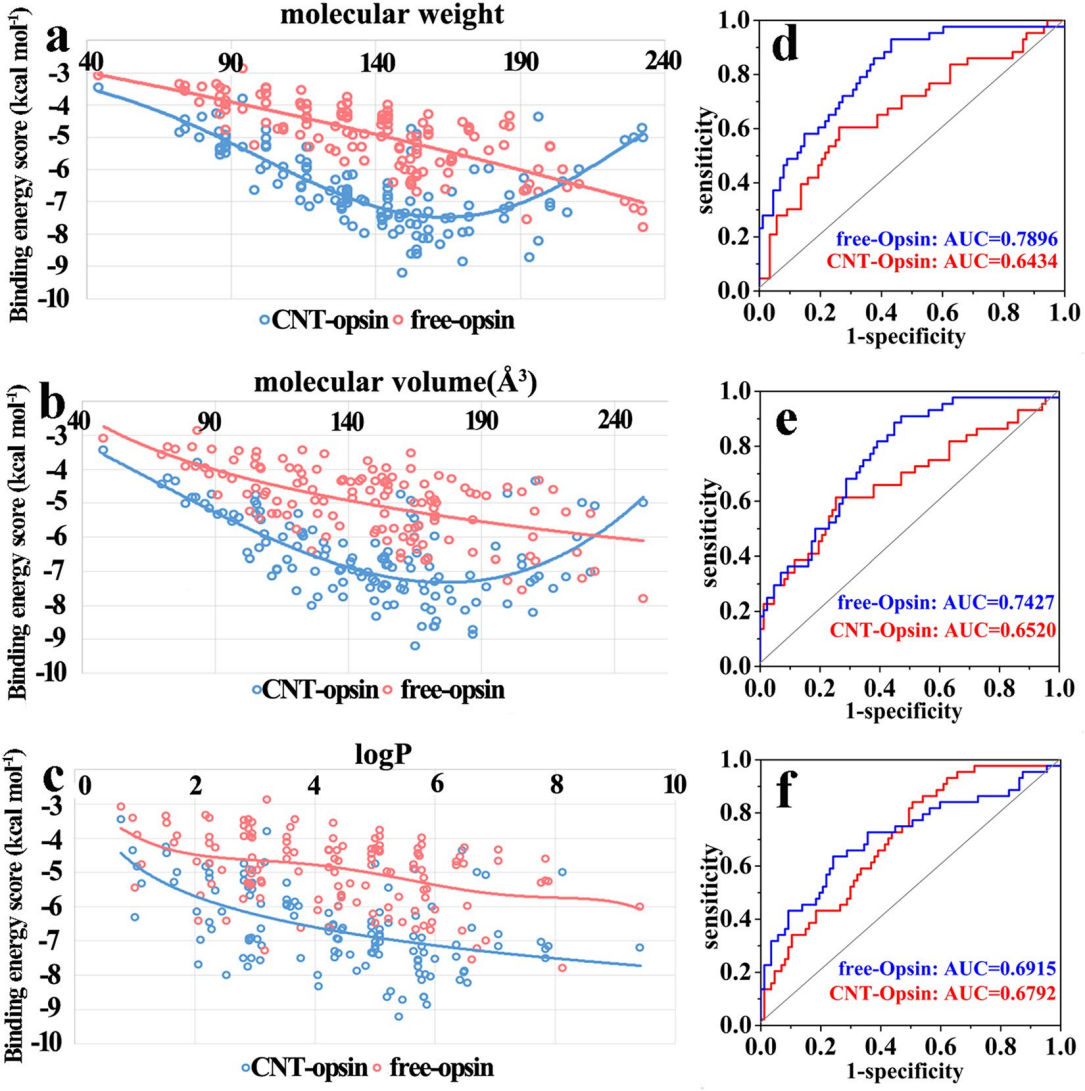
The correlations of the docking scores with three molecular descriptors and the differences in the odor-selectivity for the free and immobilized opsins. (**a**) Molecular weight, (**b**) volume, and (**c**) logP. The ROC curve of screening performance for the odors in the top one-third list from large to small for (**d**) the molecular weight, (**e**) the molecular volume and (**f**) logP value.

In order to explore the selectivity of the receptor to the different odors, we analyze the relationship between the binding energy scores and the molecular descriptors like molecular weight, volume, and logP, as depicted in Fig. 8. It can be seen from Fig. 8a and b that the correlation between the binding energy and the molecular weight is consistent with one between the binding energy and the molecular volume. For the free opsin, its affinity to the odors is strengthened with increasing of the molecular weight and volume. Similarly, the increased trend is also observed for the immobilized receptor, but only when the molecular weight and the molecular volume are less than ~160 and 170 Å^3^, respectively. When the molecular weight and the molecular volume are larger than the values, the screening performance to the odors reversely reduces with their increases. Figure 8d and e further exhibit the ROC curves for the screening performance of the opsin to 44 large odors, which are the top one-third odorants in the order list from large to small for the molecular volume and molecular weight. Judged from the AUC values in Fig. 8d,e, the free receptor is more conducive to identify the large odor molecules than the CNT-immobilized one. Previous experiments^11^ also reported that olfactory receptor OR-I7 would be activated only the odors with the 8–12 Å length and 7–11 carbon atoms in the backbone, presenting a size selectivity.

In addition, Charlie Johnson^6^ studied the response characteristics of three mouse olfactory receptor proteins expressed in heterologous surrogates and integrated them with carbon nanotube transistors for vapor response testing against eight odorants, six of which are included in our ligand set. The result from one mOR receptor (mOR 256-17) showed that the response of the receptor to some odorants are significantly influenced by the nanotube, exhibiting one change in the selectivity of the receptor to the odorant ligand. Combined with our observations, it could further confirm that the CNT-adsorption would influence the selectivity of some ORs to the odorant molecules. In terms of the change in the ligand-binding pocket upon the CNT adsorption above, we could rationalize the experimental result that the change in the selectivity of mOR256-17 should be attributed to the shrinkage of its ligand pocket induced by CNT.

**Figure 9:**
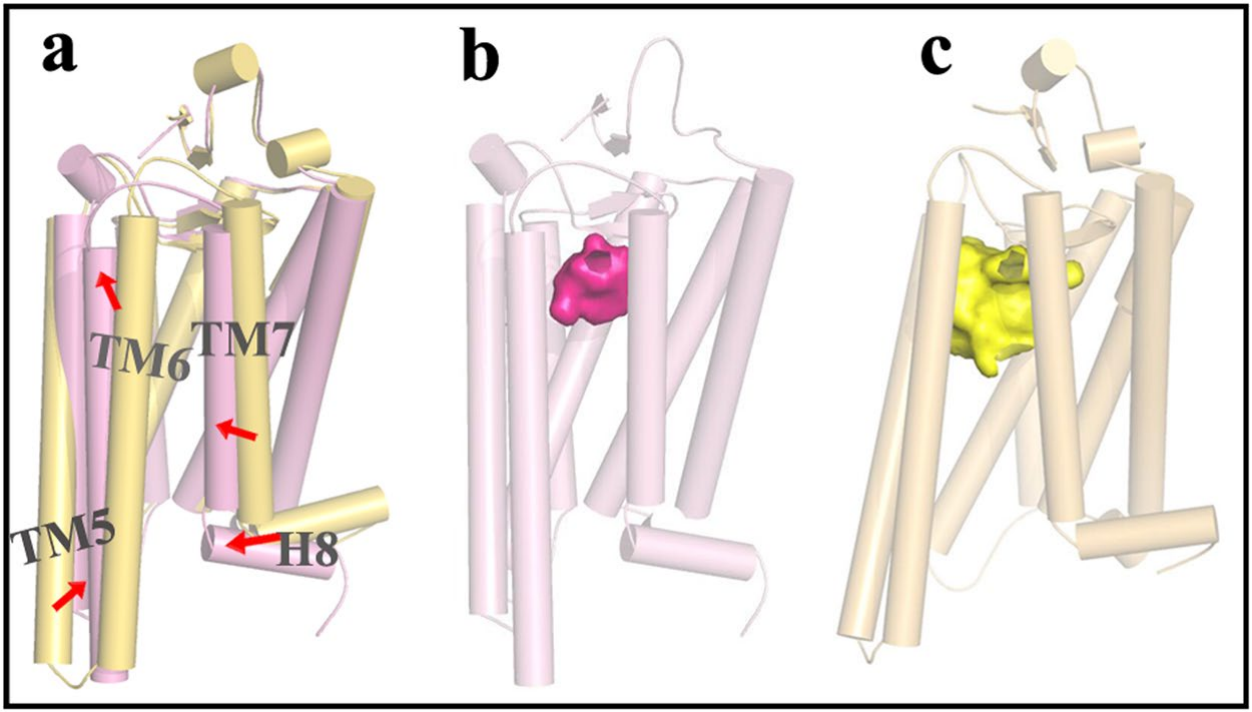
A structure superimposition and comparison between the CNT-opsin and free opsin. (**a**) The movements of transmembrane helixes (TMs) upon immobilization, derived from the comparison between (**b**) the immobilized opsin and (**c**) the free one, in which the binding pockets are displayed in surface.

To gain more insight into the size-selectivity, we further calculated the ligand binding pocket volume based on the lowest energy frame from the last nanosecond of the 1 μs trajectories for the opsin-CNT and the opsin-free systems. The pocket volumes are 320 Å^3^ for the immobilized opsin and 803 Å^3^ for the free one. The structure superimposition of the two conformations clearly shows that the CNT-adsorption could lead to inward movements of TM5, TM6 and TM7 for the opsin, leading to a significant shrinkage of the ligand binding pocket, as reflected by Fig. 9. For the free receptor, the large pocket volume could accommodate large odor molecules. Thus, its binding score is increased with increasing molecular volume. However, the narrow pocket space of the CNT-immobilized receptor only binds the molecules with proper size, thus presenting the trend with first rise and then drop. Recently, Saberi utilized mathematically statistical analysis to study the relationship between neural responses and molecular volumes of odorants for one well-structured database of odorant receptors (DoOR)^41^. Their statistical results suggested that one determinant of molecular recognition between the odorant receptors and their ligands is the molecular volume of the odorants. Our screening result further confirms the importance of the odor size in binding ORs.

LogP is the logarithm of the lipid-water partition coefficient for a compound and is an indicator of how hydrophilic or hydrophobic this compound is. As shown in Fig. 8c, with increasing hydrophobicity of the odors, the binding energies are almost increased for either the immobilized receptor or the free one. For top one-third odors (44 odors) in the hydrophobicity list from high to low, the ROC curve in Fig. 8f further shows that the free and the immobilized receptors favor to screen the 44 highly hydrophobic odors from the 132 odors, as reflected by larger AUC than 0.5. The observation provides a further support for some experimental findings that the opsins, as the photoreceptors in vision, can be activated by the ligand, retinal, which shares high hydrophobicity with OR ligands^23^.

**Figure 10:**
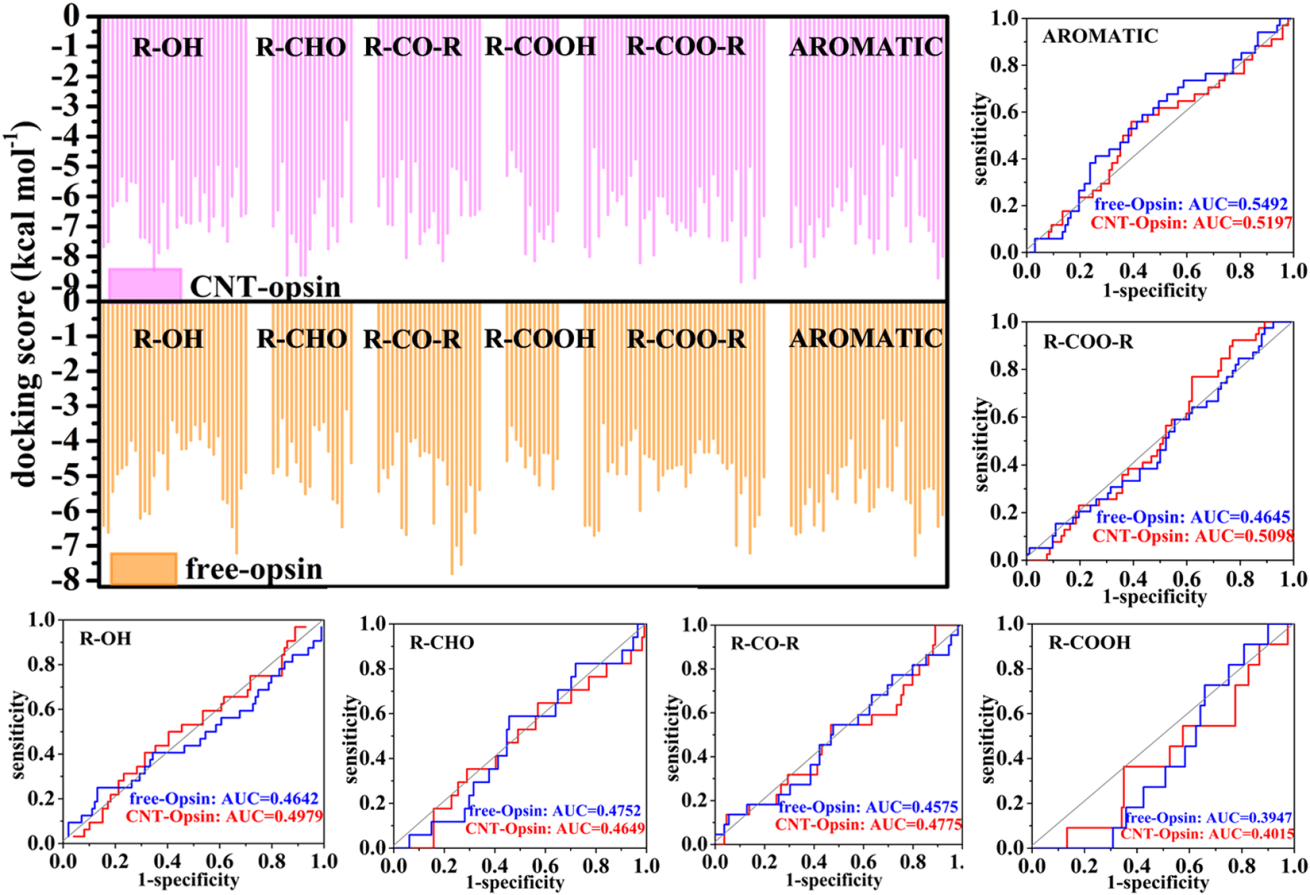
The selectivity of opsin to the odors with differently functional groups. The binding energy scores between the opsin and the odors classified by different functional groups and the ROC curves of screening performance to the odors with different functional groups for the immobilized and free opsins.

However, we could not observe a specificity of the opsin (either in the free state or in the immobilized one) to certain functional group since there is no significantly statistical difference between their selectivity to different chemical classes like alcohols, ketones, acids, esters, aldehydes, ethers, and aromatics, as evidenced by Fig. 10.

### Molecular mechanism for the size selectivity of the opsin to the odorants, derived from further MD analysis

**Figure 11:**
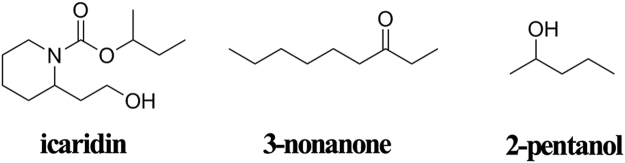
Chemical structures of the three representative odors.

As observed above, the screening of the opsin to the odors exhibits significant size-selectivity and the CNT adsorption could influence it. Thus, to explore the molecular mechanism about the size selectivity, we selected three representative odorant molecules (icaridin, 3-nonanone and 2-pentanol) to do further analysis since they present relatively high binding energy score in the docking above and are significantly different in the number of carbon atoms in the backbone (12 for icaridin, 9 for 3-nonanone and 5 for 2-pentanol), the molecular weight (229 for icaridin, 142 for 3-nonanone and 88 for 2-pentanol) and volume (228 Å^3^ for icaridin, 161 Å^3^ for 3-nonanone and 105 Å^3^ for 2-pentanol). The molecular structures of the three representative odors are shown in Fig. 11. The final docking conformations of the immobilized and free ORs with the three odors were selected to conduct further 100 ns MD simulation. The initial docking conformations and the final ones after the 100 ns simulation for the CNT-immobilized and free ORs are shown in Supplementary Fig. S1. It can be seen that the positions of the three odorant molecules are slightly changed after the MD simulation, still locating near the initial docking region. We calculated the binding free energies for the six systems under study in terms of the last 20 ns trajectory with the aid of MM/PBSA calculation, which has been considered to be a scale for assessing the affinity of ligand binding^42,43^. Table 2 lists the binding free energy and its energy components for the odor-OR complexes. As reflected by Table 2, the binding affinities of the CNT-immobilized and free opsins would drop with decreasing size of the odorants, both following the order of icaridin >3-nonanone >2-pentanol and exhibiting a conducive binding to the large-size odor. The observation is also in line with the docking result above (vide Table 2) and the experimental finding that the free olfactory-receptor has poor recognition ability to odors with the number of the carbon atoms in the backbone lower than seven^11^.

**Table 2:** Components and standard errors (in kcal mol^−1^) of the MM/PBSA binding energy between the three representative odors and the opsin in the free type and the CNT- immobilized one, which calculated using the last 20 ns trajectories of the 100 ns MD simulation. The docking scores (in kcal mol^−1^) from the virtual screening and the binding pocket volumes (in Å^3^) before the 100 ns MD simulation (labelled as V-pocket (docking)) and after that (labelled as V-pocket (after MD)).

Contribution	CNT-icaridin	Free-icaridin	CNT-3-nonanone	Free-3-nonanone	CNT-2-pentanol	Free-2- pentanol
*ΔE*_ele_^a^	−9.5 ± 3.6	−1.6 ± 0.8	−0.5 ± 0.4	0.4 ± 1.6	−2.9 ± 1.7	−2.3 ± 1.3
*ΔE*_vdw_^b^	−36.7 ± 2.4	−27.4 ± 2.3	−22.1 ± 3.2	−19.3 ± 2.1	−15.0 ± 1.6	−13.1 ± 1.4
*ΔE*_gas_^c^	−46.2 ± 4.3	−29.0 ± 3.0	−22.6 ± 2.9	−18.9 ± 2.7	−17.9 ± 2.0	−15.4 ± 2.6
*ΔG*_npsolv_^d^	−4.5 ± 0.2	−3.6 ± 0.3	−3.7 ± 0.2	−2.8 ± 0.4	−2.4 ± 0.2	−2.2 ± 0.2
*ΔG*_psolv_^e^	14.5 ± 2.9	12.6 ± 2.1	7.1 ± 1.8	6.9 ± 3.2	8.1 ± 2.1	7.4 ± 1.1
*ΔG*_solv_^f^	10.0 ± 2.8	8.9 ± 1.9	3.4 ± 0.9	4.1 ± 3.1	5.7 ± 1.6	5.2 ± 2.2
*ΔG*_binding_^g^	−26.1 ± 3.5	−20.1 ± 2.5	−19.2 ± 2.2	−14.7 ± 1.8	−12.1 ± 1.6	−10.2 ± 2.6
Docking score	−7.22	−7.62	−6.54	−4.47	−4.12	−3.86
V-pocket(docking)	320	803	320	803	320	803
V-pocket(after MD)	481	568	448	507	342	501

^a^Electrostatic energy.

^b^Van der Waals interaction energy.

^c^Total gas phase energy.

^d^Nonpolar solvation energy.

^e^Polar solvation energy calculated by solving Poisson–Boltzmann equation.

^f^Solvation energy.

^g^Binding energy.

Δ*E*_gas_ = Δ*E*_ele_ + Δ*E*_vdw_ + Δ*E*_int_, Δ*G*_solv_ = Δ*G*_npsolv_ + Δ*G*_psolv_, Δ*G*_binding_ = Δ*E*_gas_ + Δ*G*_solv_.

In addition, the binding free energies of the CNT-immobilized OR to the three odors are significantly higher than the corresponding free ones, indicating that the CNT adsorption would strengthen the affinity of the receptor to the odors (either the big size or the small one). The enhanced extents are more significant for the large odor than the small one, implying that the immobilized-opsin would be more easily activated by the large odor with respect to the free one. The observation is different from the dock results above that the adsorbed opsin presents lower affinity to the large icaridin than the free one. We assume if the difference stems from a ligand-fit effect? As shown in Table 2, the pocket volume of the opsin is increased by the CNT immobilization from 320 Å^3^ to 481 Å^3^ for icaridin, to 448 Å^3^ for 3-nonanone, and to 342 Å^3^ for 2-pentanol. For the free opsin, its volume is sharply decreased from 803 Å^3^ to 568 Å^3^ for icaridin, to 507 Å^3^ for 3-nonanone and to 501 Å^3^ for 2-pentanol. The result indicates that the binding pocket has high flexibility. The ligand-binding pocket could adjust its conformation to better accommodate and interact with the odors, presenting a significant ligand-fit binding. Due to the effect of the CNT adsorption, the pocket volume after the 100-ns MD simulation is still less for the immobilized opsin than the free one. It can be seen from Table 2 that the hydrophobic interaction (van der Waals energy) is main drive force to the odor binding for the free and the adsorbed opsins. The CNT-immobilization could enhance the van der Waals interaction through decreasing the volume of the ligand pocket. For the largest icaridin, its van der Waals interaction with the opsin is stronger than that of the two ones, leading to its stronger binding affinity than the two small ones. For the smallest 2-pentanol, the CNT-immobilization induced increase in the binding affinity is not significant with respect to the other two large odors, due to lower van der Waals interaction.

### Distribution of water molecules

**Figure 12:**
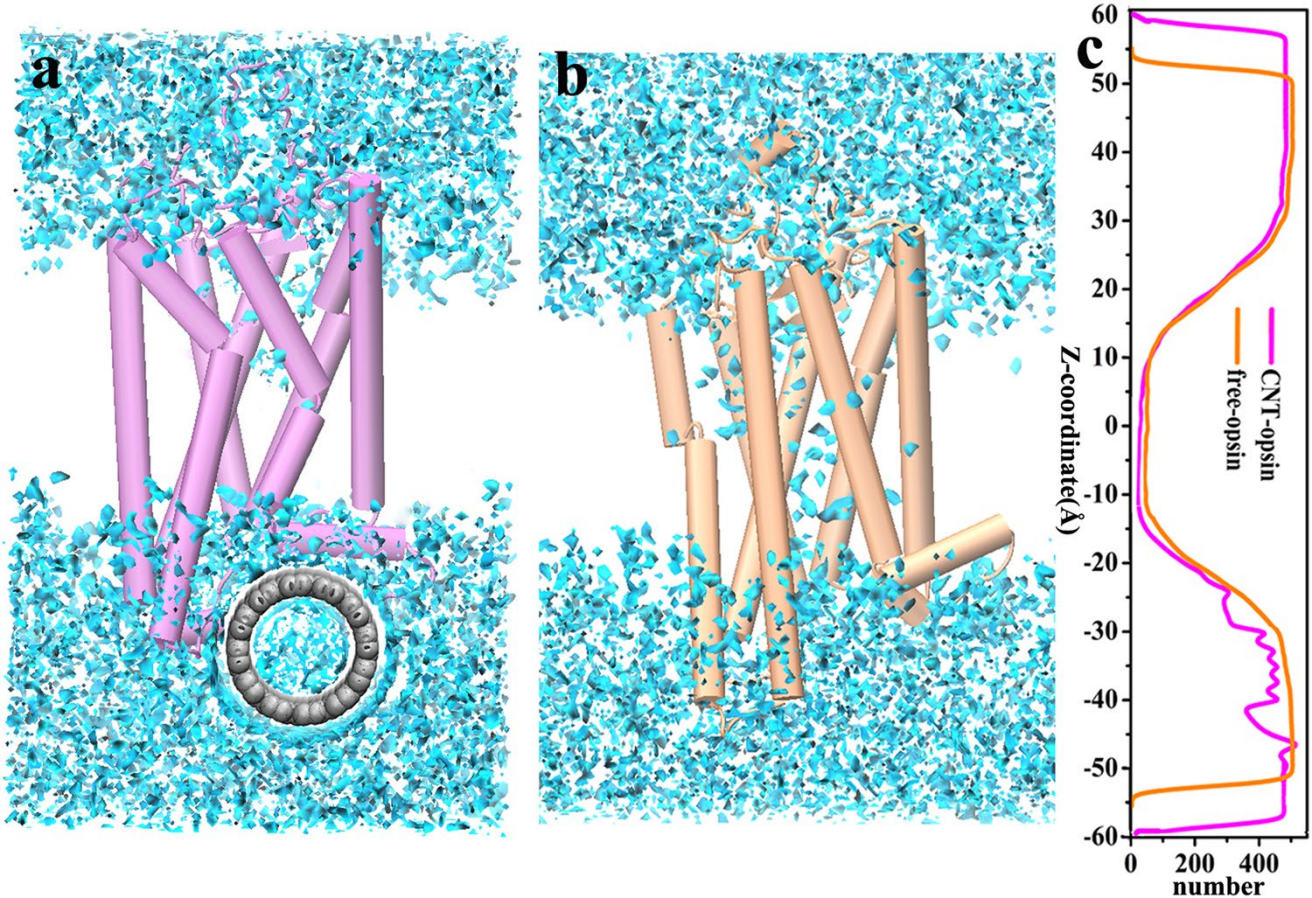
Spatial probability density distribution of the water molecules around (**a**) the CNT-immobilized opsin and (**b**) the free one. Water is displayed in blue flake. (**c**) The distribution of the number of water molecules along the Z-axis.

Since the effect of humidity on the sensitivity and selectivity of biosensor is crucial in practical application^44^, we calculated spatial probability density distribution of the water molecules around the Opsin receptor, as shown in Fig. 12. The grid densities for the solvent were binned from rms coordinate fit frames over all atoms of the receptor at 2 ps intervals for the last 100 ns trajectories of each system. We set up a grid spacing of (0.5 Å)^3^ and a box size of (150 Å)^3^ for all the calculations. The number of the water molecules along the Z-axis (perpendicular to phospholipid layer) is also shown in Fig. 12. It can be seen that the high probability regions of the water molecules are mainly located at the intracellular and the extracellular regions while the water molecules hardly enter into the interior of the hydrophobic phospholipid layer. But, some water molecules could penetrate into the interior of the receptor for the two systems, as reflected by Fig. 12a and b. Furthermore, Fig. 12c further indicates that the number of water molecules located in the interior space of the immobilized receptor is significantly less than the free system. Excessive water molecules resided at the odor-binding pocket should disturb or disfavor the ligand binding. Indeed, as observed above, the CNT-immobilization could enhance the binding energy between the odors and the receptor. In addition, it can be seen from Fig. 12a that there are some water molecules existing in the interface between the receptor and the nanotube, which should influence the adsorption since the hydrophobic van der Waals interaction is main driving force to the adsorption.

## Conclusion

With aid of the microsecond MD simulation, the protein structure network (PSN) and the virtual screening methods, we studied the effects of the CNT adsorption on the olfactory receptor structure and its selectivity to the odors, based on the two types of OR models (opsin and hOR2AG1). The results from the two models all indicate that there exists a rapid and multistep OR-adsorption onto CNT within several hundred nanoseconds, mainly driven by the hydrophobic interaction. The PSN analysis further reveals that there exist multiple allosteric communication pathways from the CNT-adsorbed residues to the odor-binding pocket, leading to the significant shrinkage of the pocket. Some important residues in the communication pathway are identified, which provide important information for the functional modification on experiments. The shrinkage of the pocket upon adsorption to some extent influences the size-selectivity of OR to the odor. For the free receptor, the selectivity to the odors is enhanced with increasing ligand size due to its large pocket space while the CNT-receptor exhibits an increase trend only for the odors with certain limited size due to its narrow pocket. When the odor size is larger than the limited value, the odor recognition is reversely weakened with increasing odor size. The screen performance of the receptor presents positive correlation with the hydrophobicity of the odor since the main drive force to bind the odors is the van der Waals interaction. In addition, our result indicates that the receptor does not exhibit observable specificity to molecular functional groups. Thus, it is the odor size and the hydrophobic property of the odor, rather than specific functional groups, that play a determinant role in binding OR, at least for the 132 odors under study. But, the 100-ns MD simulation on the complex between the receptor and the three different-size odors further reveals that the odors could induce the change in the pocket conformation to match their bindings, regardless of the limitation of the odor size in initial recognition. The result well explains the experimental controversy why an OR can be triggered by many odorant molecules and an odorant molecule can interact with different types of OR, unlike many other receptors activated only by one specific ligand. In addition, the CNT-immobilization would facilitate the receptor to bind the odors because it could enhance the van der Waals interaction through shrinking the pocket space, in particular for the large odors. The observations from the work are important for design and application of the OR-based biosensors.

## Materials and Methods

### Systems Preparation

Herein, we used the X-ray structure with 2.65 Å resolution of the active conformation of the rhodopsin apoprotein (opsin, PDB code: 4J4Q^23^), which is consisted of 326 amino acid residues and 30 crystal water molecules. The octylglucoside located in the ligand-binding pocket was removed before the MD simulation. The initial homology model of the human olfactory receptor 2AG1 with 316 residues was constructed using GPCR-ModSim^45^ webserver, based on the 2.85 Å resolution X-ray structure of rhodopsin (PDB code: 3pqr^46^). The sequence alignment of the hOR2AG1 and the rhodopsin with underlined transmembrane domains is presented in Supplementary Fig. S2. To consistent with the experimental conditions^6,9^, the lipid POPC environment and water molecules were supplemented by the CHARMM-GUI^47^ webserver (http://www.charmm-gui.org). H + + 3.0^48^ webserver (http://biophysics.cs.vt.edu/) was utilized to assign the titratable protein side chains to be the standard protonation state at pH 7.4. The single-walled CNT with chirality (12, 12) was generated via the nanotube builder plugin of the VMD software package^49^. The C-C bond length of CNT is set to be 1.42 Å and the diameter of tube is defined as about 16 Å. The length of CNT is set to be 8.0 nm, which is enough to adsorb the receptor. Following previous studie^50^, the carbon atoms of CNT were defined as uncharged Lennard-Jones particles in the simulation using sp^2^ carbon parameters. Herein, we used a clean CNT structure to represent the nanomaterial carrier, as did many computation works^51–54^. But, it should be noted that CNT could be partly oxidized in real world. Referring to experimental works^55^, we placed the ligand-binding pocket side facing opposite to the CNT so that it could effectively recognize ligands. The minimum distance between the receptor and the CNT surface is set to be 10 Å in the initial system. Counter ions K^+^ and Cl^−^ were added to keep ionic concentration at 0.15 M.

### Molecular Dynamics Simulations

All MD simulations were carried out using *pmemd.cuda* program in AMBER 16 package^56^. The ff14SB force field was used for the olfactory receptor and the lipid14 force field was used for 162 POPC molecules. The TIP3P model was adopted for water molecules. The systems were subjected to energy minimization in order to eliminate the bad contacts in the initial structures, first using steepest descent algorithm and following conjugate gradient one. Then, with aid of Berendsen-type temperature algorithm^57^, the systems were warmed up to 310 K within 120 ps, in which the carbon nanotube atoms and backbone atoms of the receptor were constrained by a 10 kcal mol^−1^ Å^−2^ position harmonic potential. After that, we performed 5-ns unconstrained MD simulations under the NVT ensemble for equilibrium. Finally, 1 μs production MD simulations with 2 fs time step were carried out under the NPT ensemble at the environment of 310 K temperature and 1 atm pressure. Coordinates were saved every 2 ps for analysis. Periodic boundary conditions were applied in all directions. The electrostatic interactions were calculated by using the particle-mesh-Ewald (PME) method^58^. All bond lengths were constrained by using the SHAKE algorithm^59^. The van der Waals interactions were treated with a cutoff distance of 12 Å. The trajectories were analyzed with the aid of the *cpptraj* module of AMBER 16 and some specific MD trajectory analysis programs.

### Protein structure network (PSN)

For the last 100-ns MD trajectories, we employed a dynamic cross-correlation (DCC) algorithm to compute the dynamical cross-correlation matrixes (DCCM) *C(i*, *j)* for the receptor Cα atoms using equation (2):

2 $$C(i,j)=\frac{c(i,j)}{c{(i,i)}^{1/2}c{(j,j)}^{1/2}}$$

where *c(i*, *j)* denotes the covariance matrix element for residues i and j.

In terms of the calculation result of DCCM, the protein structure network (PSN) method^60^ was applied to identify the shortest communication pathway between selected node pairs. In the method, the Cα atoms of residues are regarded as the nodes of the network. The edges are defined if the interaction strength I*ij* (see equation (2) below) between non-covalently interacting atoms is greater than a given interaction-strength cutoff (*I*_*min*_). Herein, *i* and *j* denote the residue identifiers, and the distance cutoff was set to 4.5 Å.

3 $${\rm{I}}ij=\frac{nij}{\sqrt{{N}_{i}{N}_{j}}}\ast 100$$

where *n*_*ij*_ is the number of distinct pairs of atoms for side chain residues *i* and *j. N*_*i*_ and *N*_*j*_ are normalization values of residues *i* and *j*, respectively, which are derived from a statistically significant protein dataset. All the DCCM and PSN calculations were performed using the Wordom^61^ MD trajectories analysis suite.

### Ligand Dataset

A set of ligands was constructed, which contains 132 odorant molecules from some theoretical or experimental works^35–38^ and involve in different chemical classes. The three-dimensional coordinates of the 132 ligands were obtained from ZINC database^62^. ChemMine Tools^63^ (http://chemmine.ucr.edu) were used to analyze and cluster these small molecules in terms of their molecular descriptors like log P, molecular weights, types and the number of functional groups and molecular volumes. The molecular volumes were calculated by using the computational chemistry software VEGA ZZ^64^.

### Virtual Screening

All the 132 odorant molecules were docked into the CNT-opsin complex and the free opsin using AutoDock 4.2 program^65^, in which the flexibility of the odorants was considered. The ligands and the receptor were then converted into the AutoDock format file (.pdbqt) by AutoDockTools. For each odorant molecule, 50 separate docking calculations were performed to ensure the accuracy of the result. We selected reasonable conformation from optimization models of the molecular docking with the lowest binding energy score to further analyze. Receiver operating characteristic (ROC) plot was applied to assess the model performance^66^, in which the farther away the curve from the diagonal, and the better the screening performance of the binary classifier system. The area under the ROC curve, which is referred as AUC, is also calculated as another indicator for the performance of the virtual screening.

### MM-PBSA calculations

Molecular mechanics Poisson Boltzmann surface area (MM/PBSA) method was used to calculate the binding free energy between two molecules, which includes molecular mechanics energy and the solvation free energy. The free energies were calculated for the complex (*G*_complex_), the receptor (*G*_recepter_) and the CNT/odor (*G*_CNT/odor_), respectively. Then, the binding free energy △*G*_binding_ was estimated in terms of following equation:

4 $${\Delta }{{G}}_{{\rm{binding}}}={{G}}_{{\rm{complex}}}-({{G}}_{{\rm{recepter}}}+{{G}}_{\mathrm{CNT}/\mathrm{odor}})$$

With the aid of the MMPBSA.py.MPI algorithm^67^ in SANDER program, the free energy G of each species was determined using the following scheme:

5 $$G={E}_{{\rm{gas}}}+{G}_{{\rm{sol}}}-TS$$

6 $${E}_{gas}={E}_{{\rm{int}}}+{E}_{{\rm{ele}}}+{E}_{{\rm{vdw}}}$$

7 $$\,{G}_{sol}={G}_{{\rm{psolv}}}+{G}_{{\rm{npsolv}}}\,$$

*E*_gas_ in equation (5) and equation (6) is the gas-phase energy, which is a sum of the internal energy (*E*_int_), the electrostatic (*E*_ele_) and the van der Waals (*E*_vdw_) energies. *G*_sol_ is the solvation free energy and can be decomposed into polar and nonpolar contributions. *G*_psolv_ is the polar solvation contribution, which could be obtained through solving Poisson-Boltzmann equation. *G*_npsolv_ denotes the nonpolar solvation contribution, which can be calculated by γ × SASA. γ represents the surface tension parameter and is set to be 0.0072 kcal Å^−2^ in this work. SASA is the solvent-accessible surface area. *T* and S in equation (5) are temperature and the total conformational entropy of the protein, respectively. Similar to many computational works^42,43^, the value of the solute entropy was not considered in the free energy calculation because we mainly focus on the change trend in the binding affinity, rather than the absolute value. The dielectric constants of the receptor interior and the external water were set to be 1 and 80, respectively. To obtain a detailed view of the interaction between the receptor and the ligand, the binding free energy decomposition to each residue was also performed using the MM-PBSA method.

## Electronic supplementary material

supplementary information
