# Supplementary material for: Shining Light on Molecular Mechanism for Odor-selectivity of CNT-immobilized Olfactory Receptor
Source: Sci Rep. 2018 May 18;8:7824. doi: 10.1038/s41598-018-26105-0 (PMC5959861; doi:10.1038/s41598-018-26105-0)
Supplement: Supplementary file 1 — supplementary information [file 41598_2018_26105_MOESM1_ESM.docx]

Supplementary Information

Shining Light on Molecular Mechanism for Odor-selectivity of CNT-immobilized Olfactory Receptor

Liyun Zhang^a^, Yuan Yuan^b^ , Tian Ren^a^, Yanzhi Guo^a^, Chuan Li^c,^*, Xuemei Pu^a,^*

^a^College of Chemistry, Sichuan University, Chengdu 610064, People’s Republic of China

^b^College of Management, Southwest University for Nationalities, Chengdu 610041, People’s Republic of China.

^c^College of Computer Science, Sichuan University, Chengdu 610064, People’s Republic of China


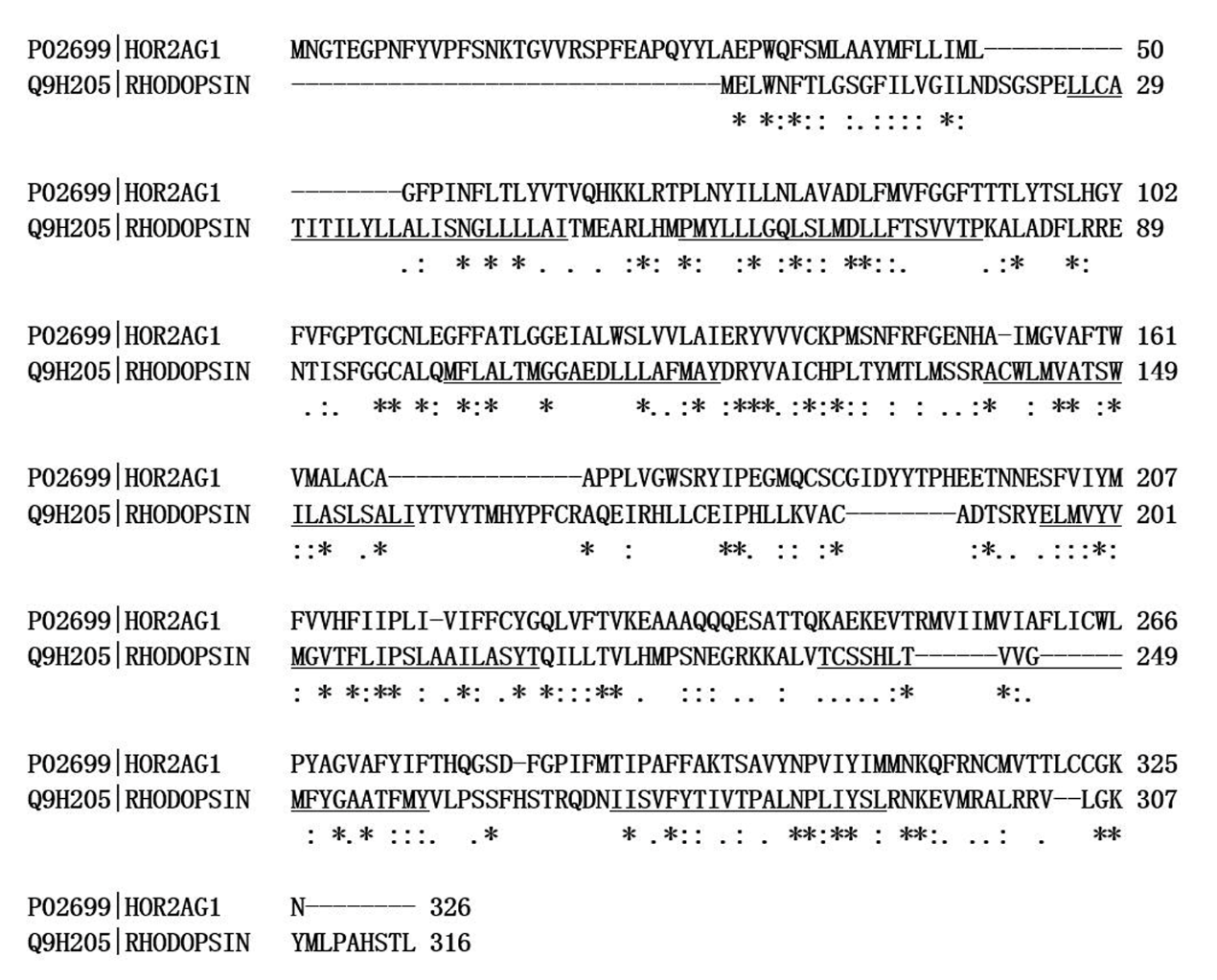


**Supplementary Figure S1. The sequence alignment of the hOR2AG1 and the rhodopsin with underlined transmembrane domains.**


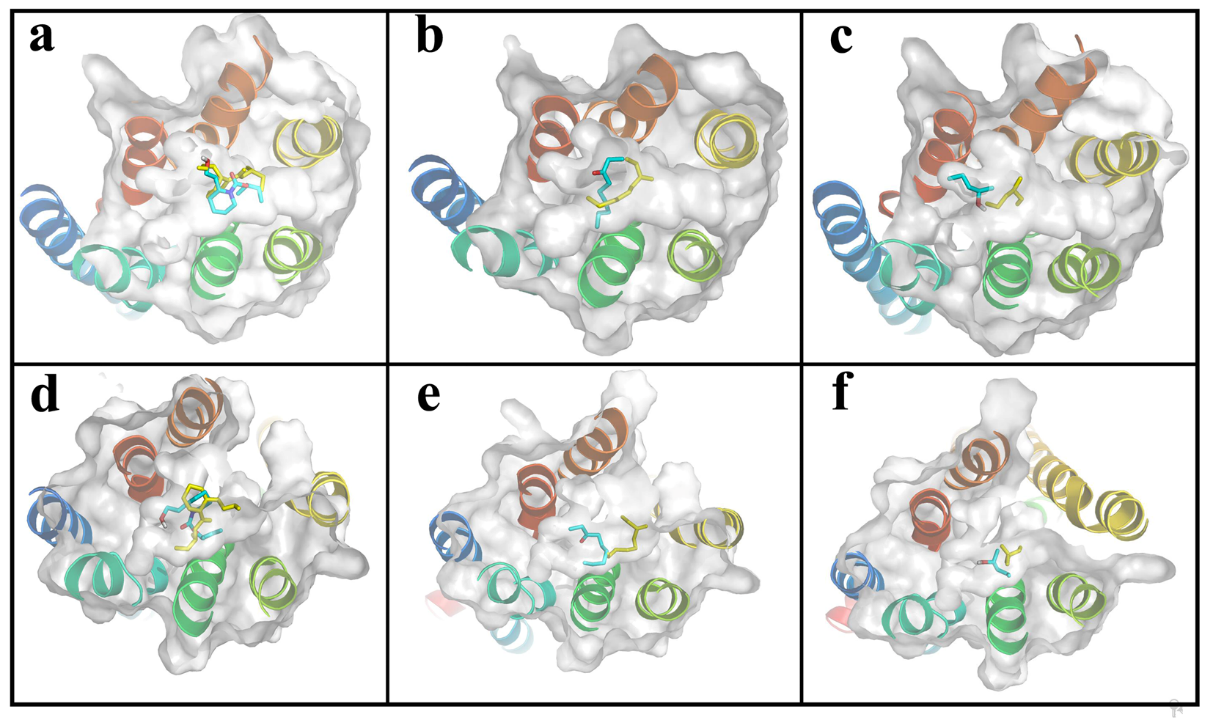


**Supplementary Figure S2. The docking conformations before and after MD simulations.** The initial odors docking conformations (colored in cyan) of (a, d) icaridin, (b, e) 3-nonanone, and (c, f) 2-pentanol and the final ones after the 50 ns MD simulation (colored in yellow) for the CNT-immobilized (top) and free (bottom) opsins.
